# Rule-Based Engine for Automatic Allocation of Smallholder Dairy Producers in Preidentified Production Clusters

**DOI:** 10.1155/2022/6944151

**Published:** 2022-06-30

**Authors:** Fatuma Mavura, Sanket M. Pandhare, Elizabeth Mkoba, Devotha G. Nyambo

**Affiliations:** Nelson Mandela African Institution of Science and Technology (NM-AIST), P.O. Box 447, Arusha, Tanzania

## Abstract

Smallholder dairy producers account for around half of all African livestock ventures; nevertheless, they face challenges in producing more milk due to an insufficient framework and infrastructure to maximize their output. Smallholder dairy producers in this scenario use a variety of tactics to boost milk output. However, the attempts need multiple heuristics, time, and financial investment. Furthermore, because of a lack of extension officers, smallholder dairy producers become trapped in failure cycles, unsuccessful attempts, and a diminished motivation to continue farming. Therefore, the interventions were more straightforward as smallholder dairy producers with comparable characteristics grouped. This research aimed to create a rule-based engine that automatically assigns smallholder dairy producers to predefined clusters. About 78 stakeholders were interviewed, including 69 smallholder dairy producers and 9 extension officers from Meru-Arusha, Tanzania. The 10 production features and 6 predefined clusters were adopted from the previous study. Therefore, a rule-based engine used the selected 10 production features. As a result, the rule-based engine automatically assigns the smallholder dairy producers to their respective clusters. Therefore, smallholder dairy producers share their farming skills and experience to increase milk output through these clusters. Furthermore, extension officers in the system provide timely assistance to smallholder dairy producers with farming concerns.

## 1. Introduction

For most farmers engaged in farming activities, smallholder dairy farming projects are a crucial source of food subsistence [1]. In Africa's livestock-farming endeavour, smallholder farmers generate nearly half of overall livestock production [2, 3]. The dairy sector has much more potential to improve livelihoods by enhancing nutrition through milk intake and increasing income from dairy product sales [1, 4]. Smallholder dairy producers face challenges in producing high milk yields to sustain their households and yet for sale. As a result, the appropriate framework and substructure have to be used to ensure that necessary services that assist them in maximizing their production are easily accessible [5]. In general, these farmers in Tanzania have cattle herds of 1–5 cows and little farms (less than 2 hectares), and they have low market preference meaning that they do not have a high value on the market [2, 3, 6]. Poor dairy farming practices such as breeding technologies, feeding practices, and proper infrastructures lead to low production and commercialization [7]. According to a recent comprehensive survey, some farmers produce significantly more than the national average (PEARL (Program for Emerging Agricultural Research Leaders) data, 2016; unpublished). According to reports, the farmers' lack of awareness of the production system they operate has deprived them of their production viewpoint in terms of marketing and yielding [8].

The fundamental characteristics of smallholder dairy producers in sub-Saharan Africa (SSA) are strikingly similar across regions [8]. Nonetheless, features relevant to farmer management techniques necessitate disaggregation. Thus, identifying specific constraints for each farm categorization, better farm management techniques, and extension services can be effective [8]. When smallholder dairy farmers work together, they learn new abilities and can better handle difficulties jointly [9]. Farmers must participate in collectiveness to get the desired outcomes [9]. Extension officers should teach smallholder dairy producers how to improve the quality and quantity of products to increase yields should be taught to farmer groups [10]. Unfortunately, there seems to be low emphasis and deprived activeness of the farmer groups (PEARL data 2016, unpublished).

Practically, heterogeneous groupings complicate service provision, knowledge exchange, and technology distribution, particularly for those who want to maximize production and profitability, due to relevant features of farmers based on management techniques [8]. Targeting dairy farmers' homogeneous groups (clusters that engage in similar managerial activities) for more accessible intervention has to be identified [11]. Smallholder dairying in homogeneous groups, where farms are classified, is well studied, sharing specific restraining causes in various farm types [12]. Extension services (such as cattle healthcare, breeding technology services, feeding technology services, and other dairy farming services) improve the quality and speed to reach smallholder dairy producers by using technologies such as mobile phones [13] if adequately designed. Electronic devices, particularly mobile phones, share information such as feeds, breeding, and health services, often requested by dairy producers [14]. In rural farming communities in sub-Saharan Africa, lack of access to information and knowledge transmission can stymie agricultural development [15].

Mobile phones give communication a new level by connecting smallholder dairy producers, accessing extension services, and raising awareness [14]. Farmers also see the cell phone as a convenient, quick, and suitable means of communication [14]. Many studies have shown the importance of social networks for farmer learning, especially peer-to-peer communication within farmer groups [16]. Various industries employ several social media networks to promote economic and production growth. People in the agriculture sector utilize social media networks to share expertise and critical information relevant to the type of activity. Users can build groups depending on their activity using social media; for example, smallholder dairy producers can form a group on any social media network and share knowledge and essential information about dairy farming. People use social media platforms such as WhatsApp, Facebook, Twitter, Clubhouse, LinkedIn, and Instagram to share information, knowledge, and experiences and express themselves [17].

In general, social media has shown to be an effective means of disseminating information about farming concerns among smallholder dairy producers (groups or individuals) and allowing farmers to share knowledge [17]. Researchers can improve smallholder dairy producers' groups by developing production clusters associated with similar characteristics. From that perspective, researchers can explore data science techniques to develop a rule-based engine that automatically allocates and groups farmers with comparable characteristics into their respective clusters. With the rule-based engine, smallholder dairy farming activities transformed from local farming (since smallholder dairy producers can interact with extension officers and get timely support) and local allocation of farmers in their groups to smart farming for increased milk output.

**Figure 1:**
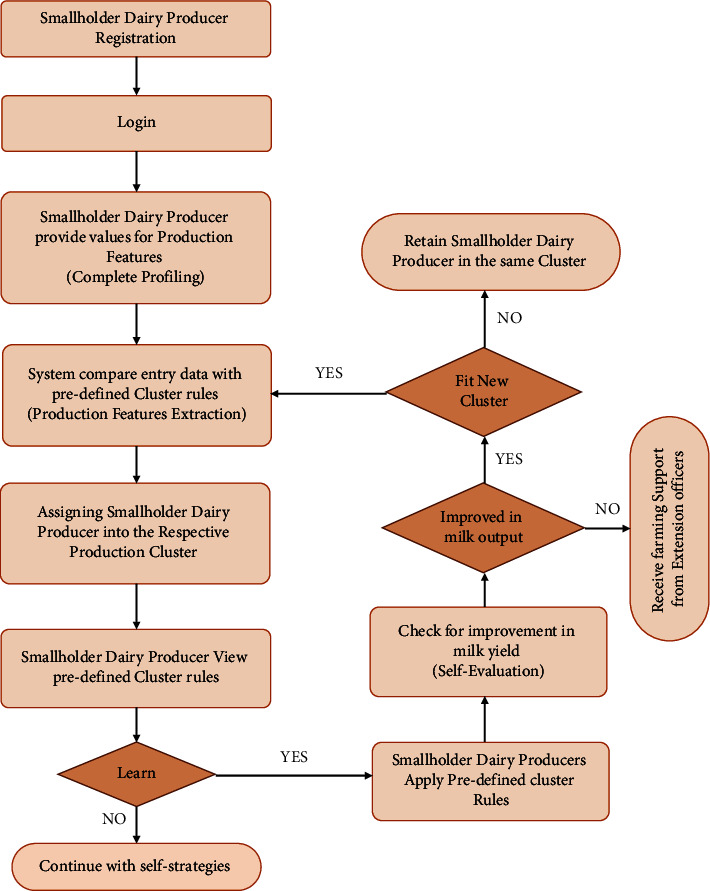
Rules used for automatic allocation of smallholder dairy producers.

Furthermore, existing systems have not taken advantage of farmers' knowledge to enhance extension support since existing systems for extension services (i.e., vaccination, extension visits, breeding technology, etc.) are centralized to support smallholder dairy producers. The study's major goal was to create a rule-based engine for automatically allocating smallholder dairy producers into specific clusters. The milk peak value is one of the attributes of clusters assignment to better their access and ability to learn from their peers to improve milk yields (see Figure 1). A rules engine is a system or software that performs actions based on specified criteria adjusted during runtime. A rule-based engine combines a collection of facts fed into the system with its rule set, causing the system to conduct one or more actions [18, 19].

Rules engine gives a computational alternative in the form of a collection of production rules with a condition and action that is as simple as an if-then statement [20]. A rule is a generalized statement such as *IF < conditions > THEN < conclusions >* [21]. Rule-based systems are an effective technology for declarative automated processing of massive data [21]. As a result, the rule-based engine allows smallholder dairy producers to share their knowledge and experience with their peers in local clusters while also receiving support from extension workers within the system. A rule-based engine is a decentralized learning approach moderated by approved extension officers and smallholder dairy producers' best milk yield.

A knowledge base holding facts, production rules, and an inference engine that manages the reasoning process make up the rules engine [21]. This study selected 10 production features for use due to their importance and relevance in dairy production. The features were vaccination frequency, watering frequency, number of milking cows, total land, litres of milk sold, frequency of extension officer visit, peak milk value, feed type, feeding frequency, and milking frequency. The allocation system is one-step toward establishing a peer-to-peer learning platform where smallholder dairy producers may interact and learn better strategies for increasing milk yield from one another. This study drew on prior peer-to-peer learning research, which used dairy production characteristics to guide cluster formation [8]. Dairy producers are grouped in specific clusters according to developed standards to learn better ways for increasing milk yield.

The developed rule-based engine is a viable and straightforward way for farmers to register by providing their production characteristics assigned to their appropriate cluster depending on the value of the data given. As a result, smallholder dairy producers in the same group can learn from one other while also learning from different clusters, resulting in higher milk yield. The following is how the article is structured: Section 2 presents Material and Methods, Section 3 presents Results, Section 4 presents Discussion, Section 5 presents Conclusions, Section 6 presents Data Availability, Section 7 presents Conflict of Interest, Section 8 presents Acknowledgements, and Section 9 presents References.

## 2. Material and Methods

### 2.1. Compilation of Data and Production Features Selection

#### 2.1.1. Compilation of Data

Standardized survey questionnaires for primary data collection to the polled smallholder dairy producers and extension officers employed. Google Form is a tool employed to create a questionnaire, and the first questionnaire was for smallholder dairy producers. The target of the survey questionnaire to smallholder dairy producers was to answer questions on activities and approaches that may help increase current milk yield. The second questionnaire was for extension officers. It aimed to determine whether any dairy farming practices mentioned above are acceptable and, when used, could boost milk yields. A total of 3,500 smallholder dairy producers in Tanzania were used as secondary data, with 6 clusters and 10 recommended production attributes from a prior study [8]. At Meru-District, Arusha (in the Kikwe, Akheri, and Sing'isi wards) was the study. Vaccination frequency, watering frequency, number of milking cows, total land, litres of milk sold, extension officer visit, feeding frequency, feed type, milking frequency, and milk peak value are among the production features. The rule-based engine employed the 10 production features in profiling smallholder dairy producers. Conversion of the gathered data (raw data) was to numeric values for analysis because the study design is quantitative; by removing incorrect data and filling in gaps, the researcher cleaned out data by deleting erroneous information and filling in blanks. Deleting unnecessary data and outliers, filling in missing values, conforming data to an assimilated pattern, and masking private or sensitive data are examples of these processes.

#### 2.1.2. Production Features Selection

The following 10 production features were selected for this study: feeding frequency, feed type, watering frequency, milking frequency, vaccination frequency, frequency of extension officer visit, milk peak value, total land, number of milking cows, and litres of milk sold. Nutritional consumption, milking frequency, high-quality feed, and ad libitum water intake are essential factors enhancing dairy cows' performance and productivity [22]. The higher the milking frequency, the better the feeding efficiency, the more frequent the feeding, and the more milk produced [22]. Dairy farmers have to examine various methods and techniques and their potential impact as they attempt to maximize herd profitability through outstanding herd performance [23]. Smallholder dairy producers can use a variety of indicators for measuring milk output, such as reproductive efficiency, cow health, and various other production attributes, to evaluate overall herd performance [23]. The feed and feeding method used influences dairy cattle productivity and reproduction. Suitable feeding is a basic need of dairy cows, and changing feeding frequency, despite appearing to be a simple idea, has been documented as a significant contributor to dairy cow production reduction [22]. Throughout lactation, dairy cows require a lot of nutrients [24]. Feeding substantial amounts of concentrates, especially during early lactation and mid-lactation, is a popular approach for meeting these high calorie and metabolizable protein (M.P.) requirements [24]. In dairy cows, reducing feeding frequency from twice to once daily reduces milk yield by 70–38%; conversely, increasing feeding frequency from twice to thrice daily increases milk production by roughly 18%, which is economically acceptable [22]. Milk output increases by 30% when the milking frequency rises from twice to trice per day. Increasing milking frequency from two to three times per day enhances overall milk output (7–20%) [22]. As a result, high-quality feed and ad libitum water, in addition to nutritional usage and milking frequency, are always significant in increasing dairy cows' performance and productivity [22]. The water needs of high-producing dairy cows are higher than that of other land-based animals. This increased demand is due to the significant amount of milk consumed, containing 87% water [25]. Water restriction had a deleterious effect on the animals in this study, as seen by animals receiving water twice a day [25]. Dairy cows have to drink enough water and eat enough feed to provide the best milk possible. Therefore, dairy cows' daily water intake and needs may be affected by the time and frequency they are watered [22]. Healthcare is one aspect of dairy cattle production that demands attention [26].

To increase our profitability, we must keep dairy cattle healthy to improve productivity [26] because the disease is one of the most severe threats to livestock production. Researchers thought that better animal health services, including training and a drug supply system that was closely monitored and supervised, as well as a more robust community disease surveillance and reporting system, would solve these issues [26]. Considering appropriate cattle healthcare delivery and disease control in these evolving farming systems is paramount given the risks posed by zoonotic pathogens and the economic consequences of disease for livestock keepers [26]. Furthermore, it is critical to establish appropriate circumstances for the rearing of dairy cows by limiting extreme climate effects such as heat and precipitation [27]. Smallholder dairy producers can reduce stress on cattle by having a good home and a well-designed farm [27]. Environmental control boosts milk production by lowering stress and disease risks while simplifying management. Safety environment in terms of suitable housing might be one of the most critical factors of production in a dairy management program [27], and therefore, 10 production features obtained.

### 2.2. Rule-Based Engine Development

**Figure 2:**
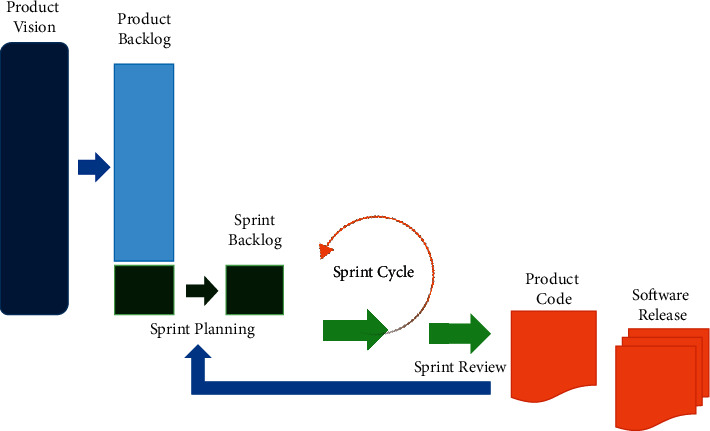
Scrum development model rule-based engine was developed based on the five stages of this model.

Android studio, visual studio code, and xampp were employed to construct the rule-based engine. Frontend employed languages such as Java and XML; the language employed for backend was PHP. MySQL is also used to handle the databases. The Scrum method, an agile software development approach, is employed in the mobile application development technique. Compared to typical waterfall methods, Scrum allows for a significant increase in productivity and reduces time to benefits [28]. In addition, scrum techniques assist businesses to quickly react to changing requirements and provide a product that aligns with developing corporate goals [28]. The Scrum method has the following steps: (a) product vision, (b) release planning, (c) product backlog, (d) sprint backlog, and (e) potentially shippable product. These procedures were used in this research (see Figure 2).

### 2.3. Characteristics of Predefined Dairy Production Clusters

This study utilized 10 production variables from [8], and the rule-based engine used the production features to profile smallholder dairy producers. After registering, smallholder dairy farmers have to complete a profile by answering 10 questions based on the 10 production characteristics. Milk peak value, feed type, feeding frequency, watering frequency, vaccination frequency, frequency of extension officer visit, a litre of milk sold, and the number of milking cows were the 10 production features employed in this study. Then, smallholder dairy producers are assigned to their respective clusters using a rule-based engine.

A rule-based engine automatically assigns smallholder dairy producers to their respective clusters. The milk peak value (scale range in litres) developed the rule-based engine. For example, if a smallholder dairy farmer produces 18 litres of milk per day, the rules engine will assign him to cluster 6 because 18 litres is within the 16–20 litres range that cluster 6 covers (see Table 1).

The table shows the characteristics used in predefined groups. Since each cluster has a varied range dependent on its performance, the developer employed the milk peak value (content) production feature to construct a backend rules engine (using mean of means). In addition, the rule-based engine checks every month to see if any smallholder dairy farmers qualify to move to a different cluster, and if so, it relocates them. The following equation shows how to calculate the mean of means:

(1) $$\begin{array}{c} \mu_{x}=\left(\frac{m1+m2+m3+\cdots +mn}{N}\right), \end{array}$$

where *µ*_*x*_ stands for the mean of means, *m* stands for means, and *N* stands for the number of means

### 2.4. Rule-Based Engine Architecture

**Figure 3:**
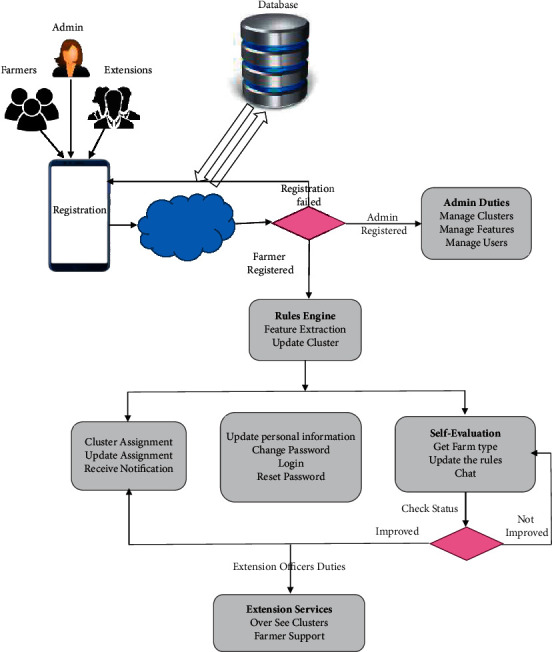
Rule-based engine architecture.

The rules engine extracts feature and assigns the smallholder dairy producer to their appropriate cluster based on the condition established after the smallholder dairy farmers finish registration. Furthermore, the smallholder dairy producer helps them learn from their peers in the same cluster by sharing knowledge and experience. A smallholder dairy producer can also assess themselves by looking at the graph of milk produced based on daily data recordings (see Figure 3).

## 3. Results

### 3.1. Performance of Clusters

The data analysis revealed the best- and low-performing clusters, as shown by each farming production feature's value. Cluster performance was measured using the 10 production features, with mean values for cluster performance in each production feature and mean means for total cluster performance. Each cluster's performance was measured for each production feature and the overall production to determine its position. The leading group has the highest mean value, whereas the following cluster has the most significant value after the top set and vice versa. To be the best performer (leading group), it should contain many smallholder dairy producers that practice particular management approaches that directly result in higher milk yield. Vaccination, feed type, feeding frequency, milking frequency, watering frequency, increased frequency of extension officer visits, and having enough total land for their cattle are all examples of production features used in this study. Furthermore, low-performing clusters have smallholder dairy producers who poorly practice those 10 production features.

**Figure 4:**
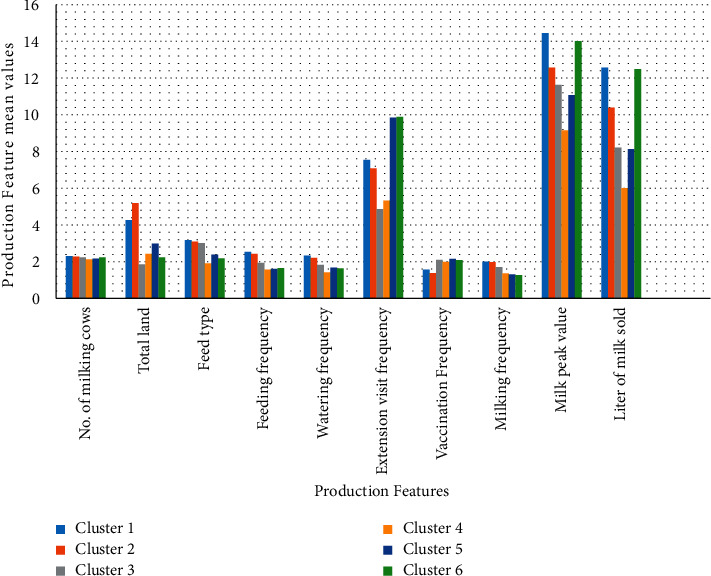
Cluster performance in specific product features.

The mean value is calculated for each group in every production feature to measure cluster performance in each production feature. The performance of each cluster in all production features is also indicated (see Table 2). The performance is determined using mean values, and a graphical depiction of the data in Table 2 is shown (see Figure 4).

This figure shows a graphical representation of each cluster's performance in every production feature. The performance of each cluster and the top-performing cluster and vice versa are depicted graphically in Figure 4. Cluster 1 leads most production features, followed by cluster 2, and then cluster 3. Cluster 4 has low performance, followed by cluster 5, and then cluster 6. The result suggests that the top cluster has a higher proportion of smallholder dairy farmers better at implementing the selected features (which indicate their management strategies) than the other clusters.

### 3.2. Position of Clusters in Each Production Feature

For the position of the cluster on each production feature, see Table 3.

This table shows groups and their position in terms of performance in each production feature. The following is how each production feature is addressed, along with its cluster position:

#### 3.2.1. Vaccination Frequency

Cluster 5 was the highest performer, followed by cluster 3, and then cluster 6, whereas cluster 4 was the lowest performer in this production feature, followed by cluster 1, and then cluster 2. Compared to the other clusters, cluster 5 had a high vaccination frequency of around a 2.15 mean value; cluster 2 had the lowest vaccination rate, with a mean value of approximately 1.37. Refer to Table 1. Smallholder dairy farmers in cluster 1 received more information about the advantages of vaccination for their livestock.

#### 3.2.2. Watering Frequency

Cluster 1 with a 2.33 mean value outperformed cluster 2 with a 2.21 mean value and cluster 3 with a 1.83 mean value in the watering frequency production feature. Cluster 4 was the lowest performer with a 1.42 mean value, followed by cluster 6 with a 1.63 mean value, and cluster 5 with a 1.67 mean value, in that order. In the watering frequency production feature, cluster 1 had a higher mean value. Therefore, smallholder dairy producers gave cattle in cluster 1 enough water than the other clusters. Cluster 1 performs better in this production aspect because most smallholder dairy producers are near water sources.

#### 3.2.3. Total Land

Cluster 2 had a higher land size for cattle keeping of about 5.18 land on average than the rest of the clusters, making it the top performer, followed by cluster 1 with an average of 4.26 land, and cluster 5 with the land of 2.98 mean value. On the other hand, cluster 3 had the lowest performance in terms of total land production feature with a mean value of 1.86, followed by cluster 6 with a mean value of 2.23, and finally cluster 4 with a mean value of 2.42.

#### 3.2.4. The Number of Milking Cows

The number of milking cows in each cluster varies slightly. Therefore, each cluster's smallholder dairy producers own nearly equal milking cows ranging from 1 to 3 milking cows. Although there is a slight difference in the number of milking cows held by smallholder dairy farmers, some have a significant milk output disparity. With this slight difference in the number of milking cows but a massive difference in milk output, it is convincing to have peer-to-peer learning since there might be smallholder dairy producers with a similar number of milking cows but differ in milk yields. Smallholder dairy producers with poor milk output can learn to improve milk yield; therefore, the mobile-based peer-to-peer learning prototype is more significant in this scenario.

#### 3.2.5. Litre of Milk Sold

Cluster 1 has a higher number of milk sales than other clusters (12.57 litres per week on average); cluster 6 came in second with high milk sales of a mean value of 12.49 litres per week. Finally, cluster 2 has weekly milk sales with a mean value of 10.39. With weekly milk sales of 6.0 litres on average, cluster 4 was the lowest-performing cluster, followed by cluster 5 with milk sales of about 8.13 litres per week. Cluster 3 was the subsequent weekly milk sales of about 8.21 litres on average. As a result, cluster 1 is the best-performing cluster, whereas cluster 4 is the lowest performer.

#### 3.2.6. Frequency of Extension Officer Visits

Cluster 6 is the most outstanding performer, with an average of 9.89 monthly extension officer visits, followed by cluster 5 with an average of 9.85 monthly extension officer visits. Cluster 1 has an average of 7.55 monthly extension officer visits. Cluster 3 had the lowest frequency of extension officer visits, at 4.86 per month, followed by cluster 4 at 5.32 per month and cluster 2 at 7.08 per month. Therefore, compared to other clusters, smallholder dairy producers in cluster 1 had a high frequency of extension officer visits for veterinary services, with an average of 9.89 trips. In contrast, smallholder dairy producers in cluster 3 had a low frequency of extension visits, with an average of 4.86 visits.

#### 3.2.7. Feed Type

Cluster 1 outperformed in this production feature with an average of 3.17 feed types, indicating that smallholder producers in this cluster fed their cattle all kinds of feed, including roughage, concentrate, and supplements, followed by clusters 2 and 3 with average feed types of 3.09 and 3.01, respectively. Cluster 5 had the lowest performance, with an average value of 2.38 feed type, followed by cluster 6 with an average value of 2.18 feed type, and then cluster 4 with an average value of 1.91 feed type.

#### 3.2.8. Feeding Frequency

Cluster 1 featured smallholder dairy producers with a high feeding frequency of 2.53 per day, followed by cluster 2 with an average of 2.42 feeding frequency per day, followed by cluster 3 with an average of 1.94 feeding frequency per day. Cluster 4 was the lowest performer in this production feature with an average of 1.65, followed by cluster 5 with an average value of 1.60 per day, and then cluster 6, which had an average value of 1.65.

#### 3.2.9. Milking Frequency

Cluster 1 had the highest performance in this production feature by having smallholder dairy producers who had a high milking frequency of about 2.0 per day, followed by cluster 2, which had an average milking frequency of 1.98 per day, and then cluster 3, which had an intermediate milking frequency of 1.70 per day. On the other hand, with an average value of 1.27 milking frequency per day, cluster 6 was the lowest-performing cluster, followed by cluster 5, which had an intermediate milking frequency of 1.31; cluster 4 had an intermediate milking frequency of 1.36 per day. Therefore, this signifies that cluster 1 has some smallholder dairy producers who milk their cows twice to trice, followed by cluster 2 in the milking frequency production feature. In contrast, most smallholder dairy farmers in other clusters milked their cows once or twice a day.

#### 3.2.10. Milk Peak Value

Cluster 1 was the top performer in this production feature by having smallholder dairy producers with a higher number of milk peak value of about 14.45 litres on average per day.

The following cluster was cluster 6 that had an average litre of 14.02 milk peak value per day and then clustered 2 with an average of 12.57 litres on average per day. The lowest-performing cluster was cluster 4 with a milk peak value of 9.15 litres per day and cluster 5 with a milk peak value of an average of 11.08 litres per day. The last was cluster 3, with an average milk peak value of 11.63 litres per day. Cluster 1 had smallholder dairy producers who produced more milk and hence had a higher milk peak value per day than the other clusters. On the other hand, cluster 4 has smallholder dairy producers with low milk yield and, therefore, low daily peak value.

### 3.3. Overall Cluster Performance for All Production Features

**Figure 5:**
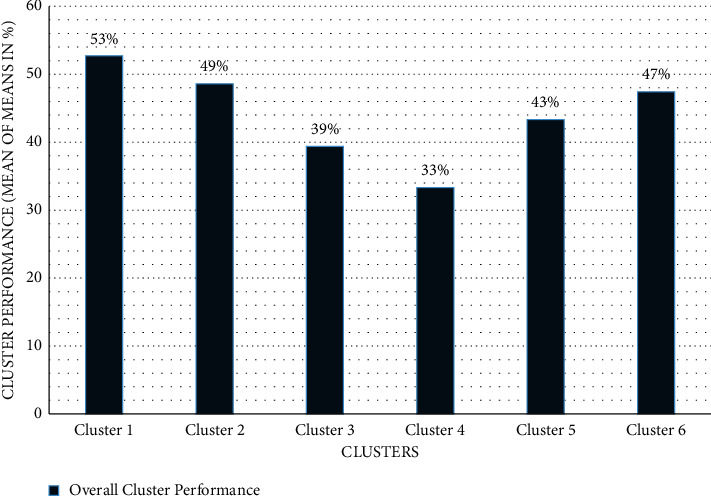
Overall cluster performance (using mean of means).

To determine the overall performance and position of the cluster based on the production features, mean of means employed. The means are the aggregate mean value of numerous samples from the same population. Cluster 1 was the top performer of all the majority of product features, with (53%), followed by cluster 2 (49%), and finally cluster 6 (47%). Because smallholder dairy producers in cluster 1 responded well to the majority (about 7 out of 10) production features, the result suggests that cluster 1 has many best-performed production features that led to higher milk yield than other clusters. Production features considered were feed type, watering frequency, feeding frequency, milking frequency, extension officer visit, and enough land for cattle keeping. Furthermore, the results suggest that cluster 5 (43%) had the lowest performance in terms of milk yield, followed by cluster 3 (39%), and finally cluster 4 (33%; see Figure 5). The results obtained suggest that the production features that directly impact milk yields, such as feed type, feeding frequency, milking frequency, vaccination, extension officer visits, and total land, are poorly practised in these lower-performing clusters.

The standard of means measures clusters' performance to the overall production features to identify the overall position of groups. Figure 5 depicts cluster performance as a whole, with smallholder dairy producers classified into clusters depending on their output in terms of milk yield. The best-performing cluster is where the best-performing smallholder dairy producers are assigned, with the following best performers assigned to the next best-performing group, and so on. The lowest-performing cluster is where lower-performing smallholder dairy producers are assigned, with the next lowest-performing farmer assigned to the lowest-performing group, and so on. The mean of means is employed to measure the performance of clusters to the overall production features; to calculate the mean of means, see equation (1).

### 3.4. Rule-Based Engine Profiling

#### 3.4.1. First Phase Profiling

The criteria for profiling are the number of milking cows, total land, extension officer visit frequency, vaccination frequency, watering frequency, feed type, feeding frequency, milking frequency, a litre of milk sold, and milk peak value. Each cluster has distinct milk peak value components (range in litres) based on its production performance and position, as shown in Table 3. The milk peak value automatically allocates smallholder dairy producers to their respective clusters using a rule-based engine. As part of a dairy farming management strategy that allows smallholder dairy farmers to learn from their peers, the other nine production features are considered dairy farming management. Smallholder dairy producers' clusters are assigned based on their milk yield (milk peak value). However, some smallholder dairy producers may encounter peers in that cluster who have the exact milk yield but differ in some of the nine production parameters of milking cows. Therefore, the rule-based engine for peer-to-peer learning became more efficient for smallholder dairy producers. The scale (range for milk peak value) used as a condition for assigning smallholder dairy producers to their respective clusters is shown in Table 4.

#### 3.4.2. Second Phase Profiling

The system does the second phase profile once a month based on the average milk yield per farmer computed using daily data entry of the litre of milk yield. Smallholder dairy farmers enter their daily milk yields into a rule-based engine to keep track of their milk production. The primary purpose of the second phase profile is to reassign the smallholder dairy producers to a different cluster based on their milk yield performance. Based on daily data input by smallholder dairy farmers, the system determines the monthly average milk yield per farmer, and smallholder dairy producers shifted to another cluster using a rule-based engine if they qualify. The rule-based engine automatically transfers the smallholder dairy producer to the appropriate cluster to meet the criteria.

### 3.5. User Registration and Login to the Prototype Implementation

**Figure 6:**
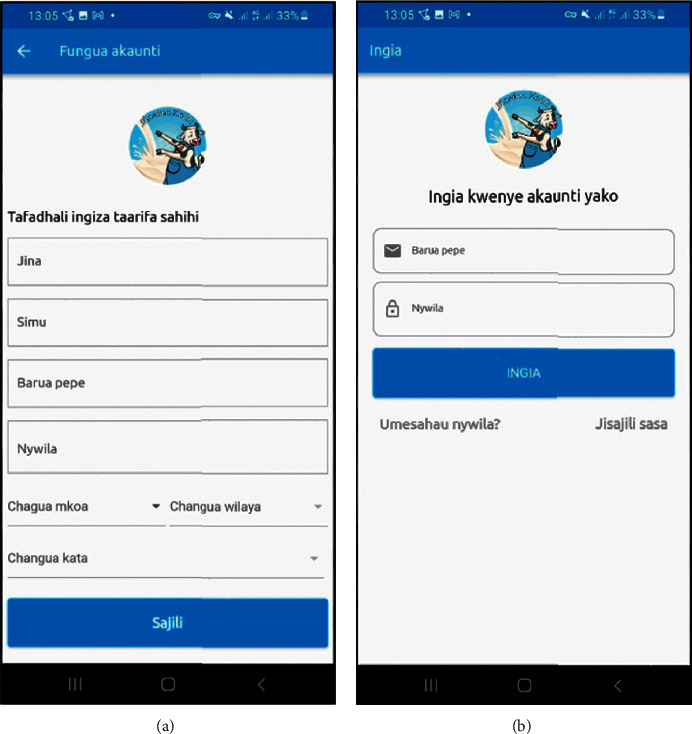
(a) The registration form for smallholder dairy producers and (b) The login form for smallholder dairy producers and extension officers.

The smallholder dairy producers have to first register to the rule-based engine by entering their name, phone number, e-mail address, password, and the region, district, and ward in which they reside, as specified on the registration form. Because most smallholder dairy producers are not familiar with English, the prototype employed Swahili for the user interface to make it easy to comprehend and operate (see Figure 6(a)). Smallholder dairy producers and extension officers have to log in to the system after completing the registration process using the e-mail and password used during the registration to continue with additional activities (see Figure 6(b)).

### 3.6. Smallholder Producer's Profiling Results

**Figure 7:**
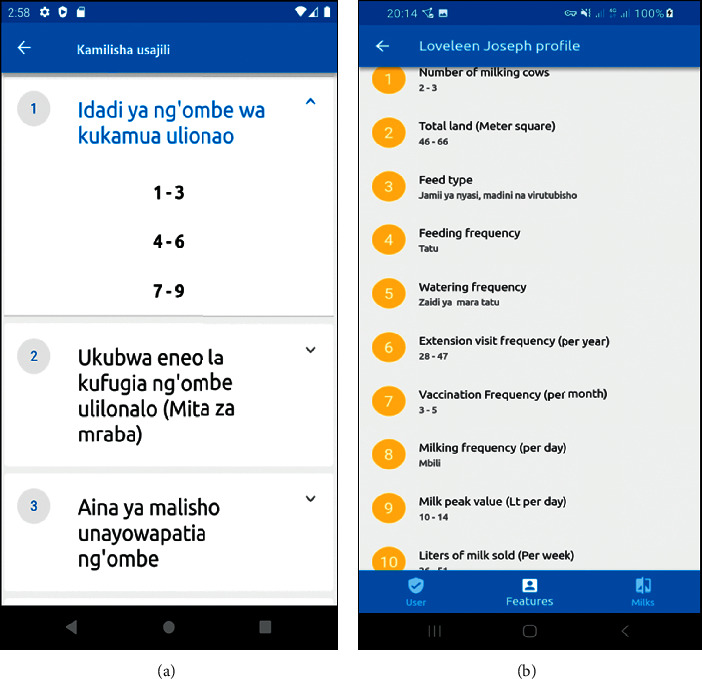
(a) A list of 10 production features in the form of questions and (b) a list of choices made by the smallholder dairy producers based on their practice in dairy farming.

Smallholder dairy producers have to complete registration by answering 10 questions (selecting replies from a list) about their operations to be assigned to one of the clusters, see Figure 7(a). Then, they offer information on how they undertake dairy farming activities by answering those ten questions. The mobile-based peer-to-peer learning prototype automatically assigns the smallholder dairy producer to their respective cluster. After completing the selection process, a smallholder dairy producer can see their options (see Figure 7(b)).

**Figure 8:**
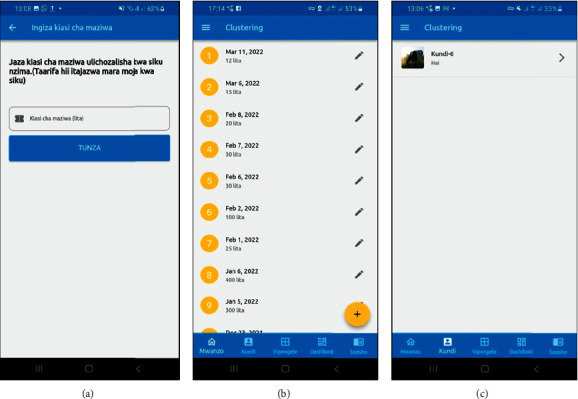
(a) The form for entering the milk yield per day in litres, (b) the records of the daily milk yield data, and (c) cluster assignment of smallholder dairy producers.

After providing entries for the features, the smallholder dairy producer has to enter the daily milk yield for cluster assignment (see Figure 8(a)). Next, smallholder dairy farmers can see all their daily milk yield statistics dating back to the first day they started entering yield data (see Figure 8(b)). Finally, the smallholder dairy producers are assigned to their respective clusters as a first phase by entering the daily milk yield. Although smallholder dairy producers clustered based on their milk yield (milk peak value), some may find their peers in that cluster who have the exact milk yield but differ in some of the nine production characteristics, such as the number of milking cows. In addition, dairy farming management varies from one smallholder dairy producer to another, such as watering frequency, feed type, and feeding frequency; therefore, this may lead to the difference in production in terms of milk yields.

Consider a smallholder dairy producer with two milking cows producing 10 litres of milk per day. You may learn from a smallholder dairy producer in the same cluster with 1 milking cow producing 10 litres of milk per day, which means peer-to-peer learning has become more efficient and significant. The system automatically assigns the smallholder dairy producer to their appropriate cluster based on the volume of milk produced every day. When the month ends after registration, the rule-based engine calculates the average milk yield for the month. If the smallholder dairy producer qualifies, it moves them to a new cluster; if not, the smallholder dairy producer stays in the same cluster (see Figure 8(c)).

**Figure 9:**
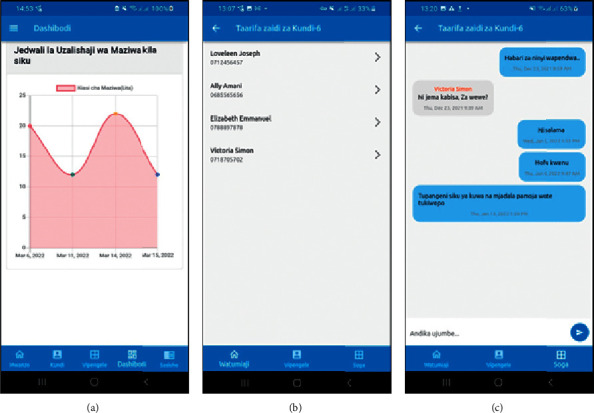
(a) The performance of smallholder dairy producers' milk yield, (b) other members of the cluster, and (c) cluster members chatting.

Smallholder dairy farmers can evaluate themselves based on daily data entry for milk yield to see the growth. Assessing the growth of smallholder dairy producers can be done by looking at their dashboards' statistical presentations (graphs; see Figure 9(a)). In addition, dairy producers from smallholder producers with comparable features are grouped in the same production cluster (see Figure 9(b)). The rule-based engine provides a chatting space for knowledge and experience sharing between smallholder dairy producers. Moreover, smallholder dairy producers can seek help from extension officers, with the primary goal of boosting milk yields (see Figure 9(c)).

## 4. Discussion

Cluster performance and rank were determined based on the findings, both for individual production features and the entire set of production features. The performance of each cluster in each production feature allowed us to see which group performed best in the majority of cases. This study evaluated the overall cluster performance to determine which group performed best across all production features. The researcher used the data to assign smallholder dairy producers to clusters based on their performance. As a result, the best-performing cluster served the best-performing smallholder dairy producers, and the rest of the producers followed orders depending on their performance.

Rules generated using the milk peak value automatically allocate smallholder dairy producers to their appropriate clusters because of the successful development of a rule-based engine for the automatic allocation of smallholder dairy producers. Many studies have shown the importance of social networks for farmer learning, especially peer-to-peer communication within farmer groups [16]. Through the rules-based engine, smallholder dairy producers can learn from their peers by sharing information and experience through conversing about farming concerns within their clusters, all to enhance milk output, according to the built rule-based engine. Moreover, small-scale dairy farmers can assess their performance in terms of milk yield by viewing a graph generated from daily milk yield data entering.

Agricultural extension and advising services are critical agents in agricultural development, poverty alleviation, and food security [29]. Agricultural extension and advisory services can help farmers address production and management issues by improving their technical knowledge, farm management skills, and information systems, resulting in increased production, higher economic returns, and a boost to the national and global economies [29]. Extension services and delivery techniques, on the other hand, are often unsuccessful in meeting the needs and technological hurdles that farmers face [29]. Moreover, farmers share their knowledge, bridging the gap between them and extension officers [30].

Smallholder dairy farmers can get timely farming advice from extension officers and receive various updates in their clusters using a rule-based engine. These updates are subjects to managing a dairy farm to achieve high milk production. As a result, the rule-based engine simplified knowledge sharing and experience regarding farming. Extension support for veterinary services has reached smallholder dairy producers promptly by simply contacting the extension officer within their clusters. This study used smallholder dairy producers with experience in that area, and excellent milk yields augmented extension officers.

## 5. Conclusions

The primary purpose of this research was to create a rule-based engine that would automatically assign smallholder dairy farmers to predefined production clusters depending on milk output performance. The purpose of giving smallholder dairy farmers to their respective predefined production clusters was to group smallholder dairy producers with comparable features to share knowledge and experience about farming concerns with the primary goal of increasing milk yield. According to the findings of this study, the rule-based engine automatically assigned smallholder dairy producers to their respective clusters. As a result, smallholder dairy farmers in these clusters exchange their agricultural knowledge and experience, interact with extension agents, and receive timely assistance depending on their needs in dairy farming. Moreover, suppose a smallholder dairy producer meets the performance criteria based on the milk yield of another cluster. In that case, the rule-based engine automatically shifts that smallholder dairy producer to that cluster. Furthermore, smallholder dairy producers were able to self-evaluate by adopting agricultural approaches from their peers based on cluster production features and updates supplied by the system's extension officers. Smallholder dairy producers can also monitor the performance of their daily milk yields by looking at a graph of milk yields presented on their dashboards.

## Figures and Tables

**Table 1:** Characteristics of predefined dairy production clusters.

Cluster	Mean of means (%)	Production features	Milk peak value scale (range in litres)
1	53	Milk peak value, feed type, feeding frequency, watering frequency, vaccination frequency, frequency of extension officer visit, a litre of milk sold, number of milking cows	26–30
2	49	Milk peak value, feed type, feeding frequency, watering frequency, vaccination frequency, frequency of extension officer, a litre of milk sold, number of milking cows	21–25
3	39	Milk peak value, feed type, feeding frequency, watering frequency, vaccination frequency, frequency of extension officer, a litre of milk sold, number of milking cows	6–10
4	33	Milk peak value, feed type, feeding frequency, watering frequency, vaccination frequency, frequency of extension officer visit, a litre of milk sold, number of milking cows	1–5
5	43	Milk peak value, feed type, feeding frequency, watering frequency, vaccination frequency, frequency of extension officer, a litre of milk sold, number of milking cows	11–15
6	47	Milk peak value, feed type, feeding frequency, watering frequency, vaccination frequency, frequency of extension officer, a litre of milk sold, number of milking cows	16–20

**Table 2:** All the production features in all clusters with their values.

Production features	Cluster 1	Cluster 2	Cluster 3	Cluster 4	Cluster 5	Cluster 6
	Mean value	Mean value	Mean value	Mean value	Mean value	Mean value
Vaccination frequency	1.56	1.37	2.10	1.99	2.15	2.08
Watering frequency	2.33	2.21	1.83	1.42	1.67	1.63
No. of milking cows	2.30	2.27	2.24	2.13	2.17	2.23
Total land	4.26	5.18	1.86	2.42	2.98	2.23
Litre sold	12.57	10.39	8.21	6.0	8.13	12.49
Frequency of extension officer visit	7.55	7.08	4.86	5.32	9.85	9.89
Milk peak value	14.45	12.57	11.63	9.15	11.08	14.02
Feed type	3.17	3.09	3.01	1.91	2.38	2.18
Feeding frequency	2.53	2.42	1.94	1.57	1.60	1.65
Milking frequency	2.00	1.98	1.70	1.36	1.31	1.27

**Table 3:** Position of the cluster based on its score on each production feature.

Production features	Position of the clusters					
	Position 1	Position 2	Position 3	Position 4	Position 5	Position 6
Vaccination frequency	Cluster 5	Cluster 3	Cluster 6	Cluster 4	Cluster 1	Cluster 2
Watering frequency	Cluster 1	Cluster 2	Cluster 3	Cluster 6	Cluster 5	Cluster 4
No. of milking cows	Cluster 1	Cluster 2	Cluster 3	Cluster 6	Cluster 5	Cluster 4
Total land	Cluster 2	Cluster 1	Cluster 5	Cluster 4	Cluster 6	Cluster 3
Litre sold	Cluster 1	Cluster 6	Cluster 2	Cluster 3	Cluster 5	Cluster 4
Extension visit	Cluster 6	Cluster 5	Cluster 1	Cluster 2	Cluster 4	Cluster 3
Feed type	Cluster 1	Cluster 2	Cluster 3	Cluster 5	Cluster 6	Cluster 4
Feeding frequency	Cluster 1	Cluster 2	Cluster 3	Cluster 6	Cluster 5	Cluster 4
Milking frequency	Cluster 1	Cluster 2	Cluster 3	Cluster 4	Cluster 5	Cluster 6
Milk peak value	Cluster 1	Cluster 6	Cluster 2	Cluster 3	Cluster 5	Cluster 4

**Table 4:** Milk peak value scale (range) for cluster assignment.

S/No	Cluster	Mean of means (%)	Milk peak value scale (range in litres per day)
1	Cluster 1	53	26–30
2	Cluster 2	49	21–25
3	Cluster 6	47	16–20
4	Cluster 5	43	11–15
5	Cluster 3	39	6–10
6	Cluster 4	33	1–5

## Data Availability

Upon request, the corresponding author will provide the data that support the conclusions of this study.
